# Comprehensive dataset of temperature and strain-rate dependent material behavior of HCT590X and EN-AW 6014

**DOI:** 10.1016/j.dib.2026.113245

**Published:** 2026-09-11

**Authors:** Johannes Gerritzen, Malte Schlichter, Stefan Goetz, Robert Kupfer

**Affiliations:** a TUD Dresden University of Technology, Institute of Lightweight Engineering and Polymer Technology (ILK), Holbeinstr. 3, Dresden, 01307, Germany; b Paderborn University, Laboratory for Material and Joining Technology (LWF), Pohlweg 47, Paderborn, 33098, Germany; c Friedrich-Alexander-Universität Erlangen-Nürnberg, Chair of Engineering Design, Martensstraße 9, Erlangen, 91058, Germany

**Keywords:** Material behavior, Strain-rate dependency, Temperature dependency, Stress-strain curves, Tensile Testing

## Abstract

The material behavior of two metals with high usage rates in the automotive sector was investigated: The dual-phase steel HCT590X and the hardenable aluminum EN-AW 6014 in T4 as well as T6 state. For this, tensile tests were conducted at various strain rates and temperatures. Local strain was determined by digital image correlation in all tests. Force was measured by the load cell in quasi-static tests and derived from the calibrated dynamometric specimen section in high-strain-rate tests. Longitudinal stress was calculated using specimen cross-sectional area. A total of 66 raw force-displacement- and derived stress-strain curves are provided for the HCT590X and 123 for the EN-AW 6014. They are enriched with the necessary metadata to allow mapping with established ontological frameworks at different levels, namely metadata4ing (provenance and process), Platform Material Digital: Core Ontology (process and property descriptions) and Tensile Test Ontology (ISO 6892-based representation). The stress-strain data is well-suited for calibrating and validating constitutive models including strain rate and temperature dependency or to enlarge a topic specific knowledge graph.

Specifications Table

**Table:** 

Subject	Engineering & Materials science
Specific subject area	Stress-strain behavior of HCT590X and EN-AW-6014 at various strain rates and temperatures
Type of data	Table, Image, Chart, Graph, Figure. Raw, Analyzed, Processed
Data collection	Tensile tests at different temperatures and strain rates were conducted for the dual-phase steel HCT590X and hardenable aluminum EN AW-6014. An Instron VHS 65/80–20 servo-hydraulic test machine was used for high-strain-rate experiments, deformation was obtained from 2D DIC measurements with stochastic gray scale pattern. Else, a Zwick Z100 test machine with GOM ARAMIS system for strain measurement was used. For the quasi-static tests, force was measured using the load cell of the testing machine. For the high-strain-rate tests, force was determined from the calibrated strain gauge signal of the dynamometric specimen section according to SEP 1230. In both cases, engineering stress was calculated from the respective force signal and the initial specimen cross-sectional area.
Data source location	All experiments were conducted in Germany at Paderborn University, Laboratory for Material and Joining Technology (LWF).
Data accessibility	Repository name: Zenodo Data identification number: https://doi.org/10.5281/zenodo.21390267 Direct URL to data: https://zenodo.org/records/21993433
Related research article	The presented data has been part of 32 publications to date.

## 1 Value of the Data

• Comprehensive collection of experimentally determined, high-fidelity stress-strain curves of HCT590X steel and EN-AW 6014 aluminum, materials frequently used in automotive industry, under various temperature and strain rate conditions.
• Stress-strain curves can be used to calibrate and validate constitutive models for HCT590X steel and EN-AW 6014 aluminum across a broad range of conditions. These models may subsequently be used as basis for process or structural simulations.
• Material properties relevant for dimensioning, such as yield stress or ultimate tensile strength, as well as their dependency on temperature and strain rate can be extracted from the provided data.
• Data can be used to validate new testing equipment or to enrich other datasets, improving their statistical significance
• Comprehensive, ontology ready set of metadata allows for mapping with knowledge graphs

## 2 Background

With the continuous increase in importance of non-linear analyses in designing and dimensioning structural components, the importance of high-fidelity experimental data for the material behavior rises. However, despite the variety of academic papers and other works relying on accurate material models, determining the underlying material data remains a typical prerequisite for such developments. The reasons for this are manifold, but can often be traced back to lack of findability, insufficient documentation of data acquisition processes or the publication of aggregated data that significantly limit their re-usability. This delays the development of novel models and inevitably leads to sub-optimal usage of resources, since existing tests have to be repeated. Therefore, the goal of this work is to establish a dataset of reliable material data [1] with a comprehensive documentation of the acquisition process and robust findability via ontology-ready labeling. This will be presented exemplary for two material systems of high relevance in the automotive sector: a dual-phase steel HCT590X [2], and a heat treatable aluminum EN AW-6014 [3], both with high importance for the automotive sector [4], thus increasing the availability of material data to efficiently tackle development challenges in the field of engineering and providing a blueprint for future publication of material data for mechanical engineers, ensuring compliance with the FAIR-principles [5].

## 3 Data Description

To ensure that the published data can be efficiently found, interpreted, and reused, the dataset is accompanied by a structured metadata schema that follows the FAIR principles [5]. The schema has been developed as a conceptual model that covers the complete path from the overall dataset down to individual files and is aligned with current efforts in research data management in materials and engineering sciences, such as those driven by NFDI-MatWerk [6] and related initiatives.

The metadata schema distinguishes three levels. On the dataset level, it captures descriptive information such as title, abstract, contributing authors, institutional affiliations, associated projects and subprojects, version, license, and links to related publications or software artifacts. On the experiment and specimen level, the schema records the material and its condition (e.g., alloy designation, sheet thickness, heat treatment), specimen geometry, and identifiers, as well as all relevant test conditions. This includes the testing machine and sensor systems, control mode, strain rate, temperature, applied standards, and information on the measurement setup. On the file and technical level, each data file is linked to its corresponding series of experiments and is annotated with file type and format, processing state (raw vs. processed), sampling rates and units.

The schema is intentionally designed to be ontology-ready rather than a standalone, project-specific vocabulary. On the provenance and process side, its concepts can be mapped to the Metadata4Ing ontology [7], which provides a generic framework for describing research workflows and data generation processes in engineering. For material, process, and property descriptions, the schema follows the modeling patterns of the Platform MaterialDigital Core Ontology (PMDco) [8] as a mid-level ontology for materials science and engineering. Finally, the tensile-test-specific metadata has been structured to be compatible with the Tensile Test Ontology (TTO) [9], which offers a standard-compliant, ISO 6892-based representation of tensile tests and their results. While the current work employs this schema primarily as a conceptual backbone for consistent metadata, its ontological alignment prepares the data for future integration into knowledge-graph-based infrastructures and NFDI-level services.

**Fig. 1:**
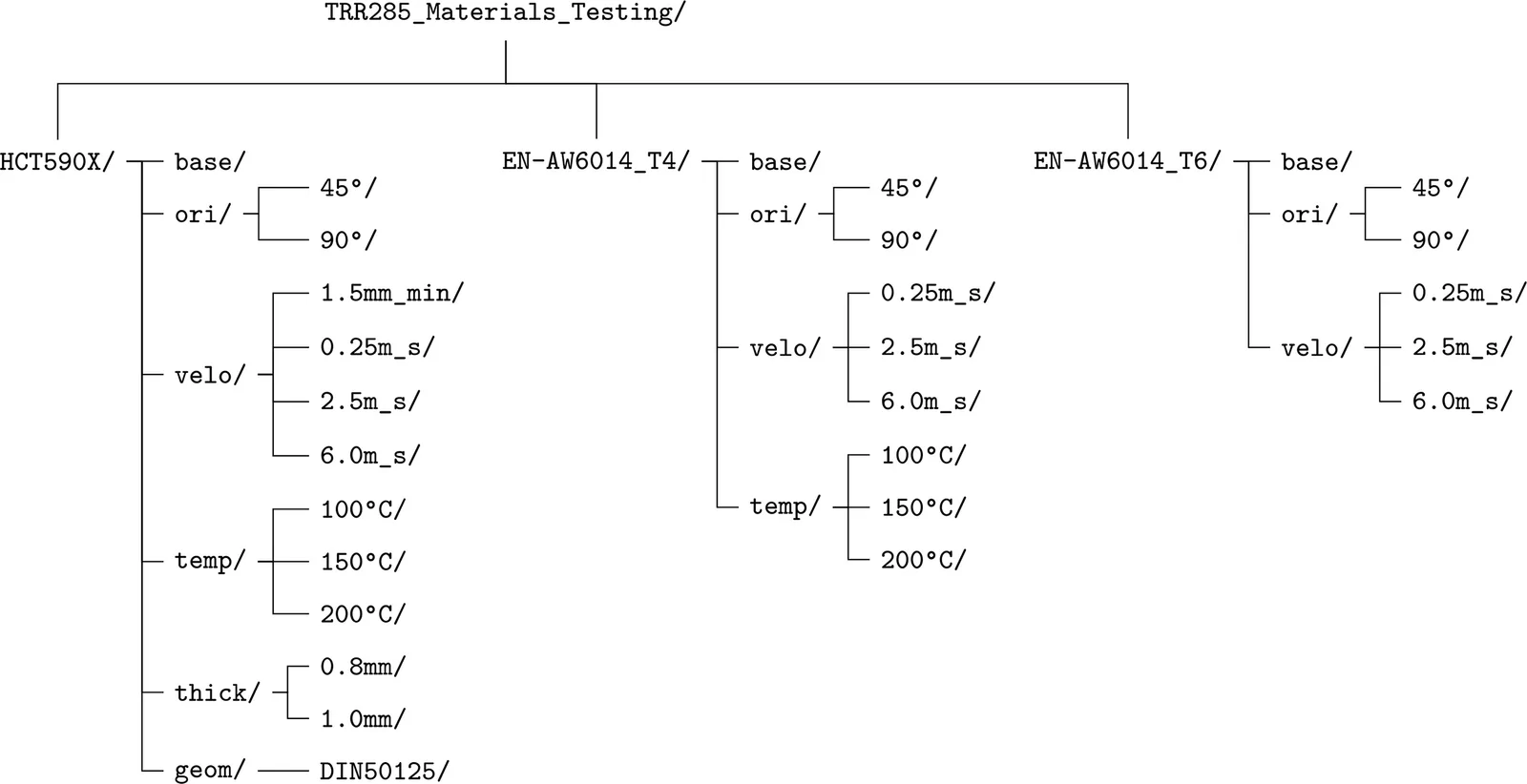
Schematic folder structures for conducted experiments.

The data is stored in a hierarchical folder structure. At the top level, materials are distinguished i.e., HCT590X, EN-AW6014-T4, and EN-AW6014-T6. For each material, a base configuration of tested variations is defined to 15 mm/min cross-head velocity, 23 °C test temperature, 0° orientation with respect to the sheet rolling direction (RD), and a sheet thickness of 1.5 mm for HCT590X and 2.0 mm for EN-AW6014. The base folder contains an Excel file with raw force and displacement, as well as derived strain and stress data for all specimen tested under this configuration. Additionally, a zip archive containing images taken throughout the test, and raw ARAMIS measurements, where applicable, is present. Data obtained by deviating from the base test setup are structured similarly: an excel sheet containing raw force-displacement, and derived stress-strain data and a zip archive with raw supplementary data. A detailed overview of investigated factor combinations is given in Table 1, schematics of the resulting folder structure in Fig. 1. To facilitate interoperability with ontologies and programmatic analyses, exemplary mapping files for key measurement data are provided as well.

**Table 1:** Overview of the investigated factor combinations and respective number conducted tests.

Varied factor	Levels	HCT590X	EN-AW6014 T4	EN-AW6014 T6
– (baseline)	–	5	5	3
Orientation to RD	45° / 90°	5 / 5	5 / 5	3 / 3
Strain rate	0.001 / 10 / 100 / 240 s⁻¹	3 / 3 / 3 / 3	– / 5 / 5 / 5	– / 5 / 5 / 5
Orientation × strain rate	(45°, 90°) × (10, 100, 240 s⁻¹)	–	5^a^ (30)	5^a^ (30)
Temperature	100 / 150 / 200 °C	3 / 3 / 3	3 / 3 / 3	–
Sheet thickness	0.8 / 1.0 mm, each at 0° and 90°	5^a^ (20)	–	–
Specimen geometry	DIN 50125, 1.0 / 1.5 mm thickness	5 / 5	–	–
**Total**		**66**	**69**	**54**

a Specimen number per level; total number tested in brackets.

**Fig. 2:**
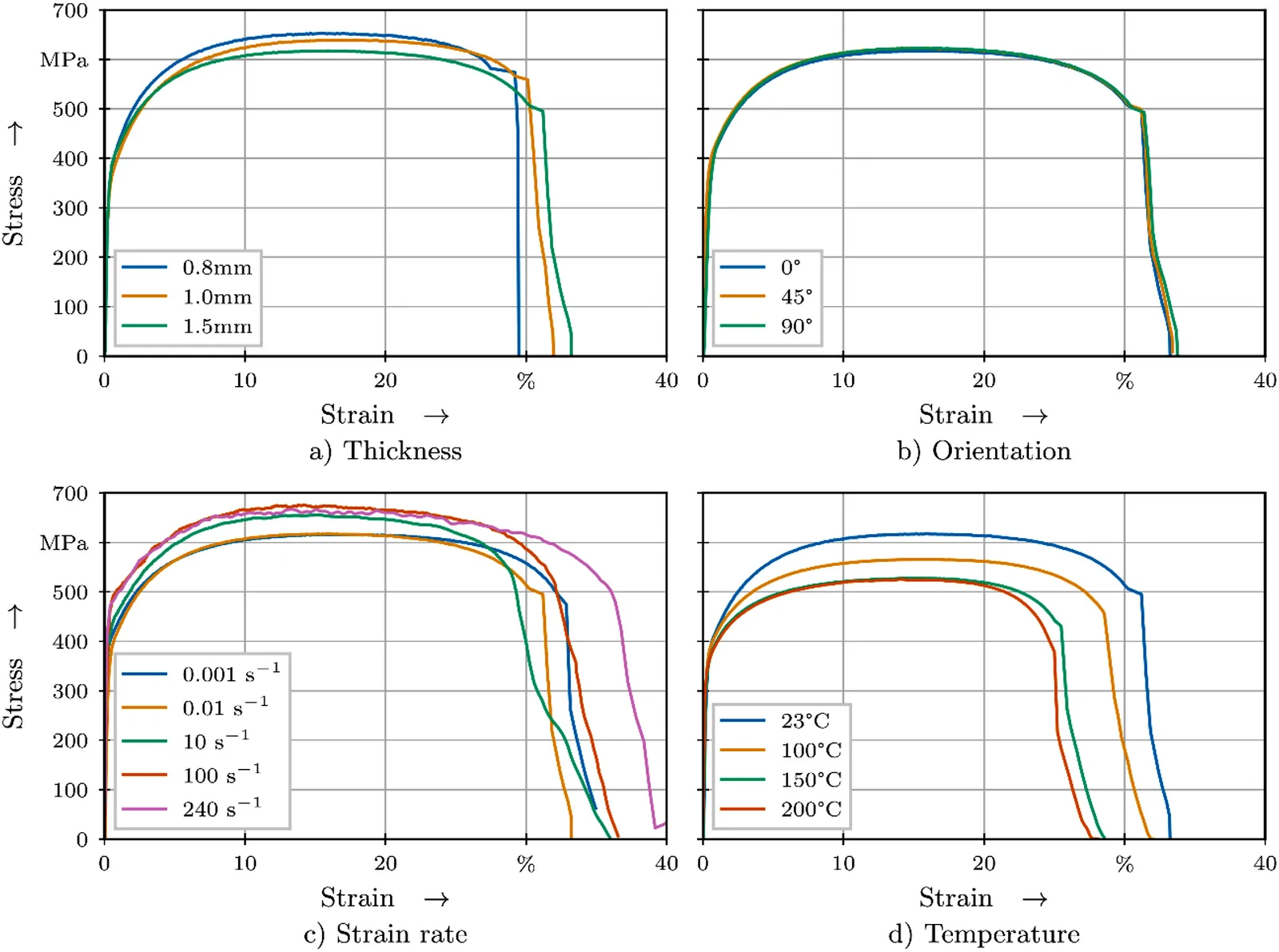
Representative stress-strain curves for different variations in testing conditions of HCT590X.

Material behavior is typically analyzed by their stress-strain relationship, removing the influence of geometry. Similarly, rate of deformation is characterized by the nominal strain rate $\dot{\varepsilon }$ per

(1) $$\dot{\varepsilon }=v_{0}/l_{p}$$

with cross-head velocity $v_{0}$ and parallel test length $l_{p}$ [10]. Representative curves for HCT590X are given in Fig. 2, illustrating influence of sheet thickness (a), specimen orientation with respect to RD (b), strain rate (c), and temperature (d). From this it becomes clear that limit stress decreases slightly with sheet thickness, whereas total elongation increases. Both are unaffected by the specimen’s orientation. With regard to strain rate, two regions of transition and level of the stress plateau may be identified, one at low rates slightly exceeding 600 MPa and the other at moderate rates reaching 670 MPa. Here, no clear trend in total elongation is present. Increase in temperature leads to the most pronounced changes in material behavior. Only the linear region, up to the transition to non-linear behavior, remains unaffected. The stress plateau significantly decreases up to 150 °C, as does the transition speed. Further increase to 200 °C does not lead to further reduction in limit stress. Total elongation, on the other hand, continuously decreases with increasing temperature.

**Fig. 3:**
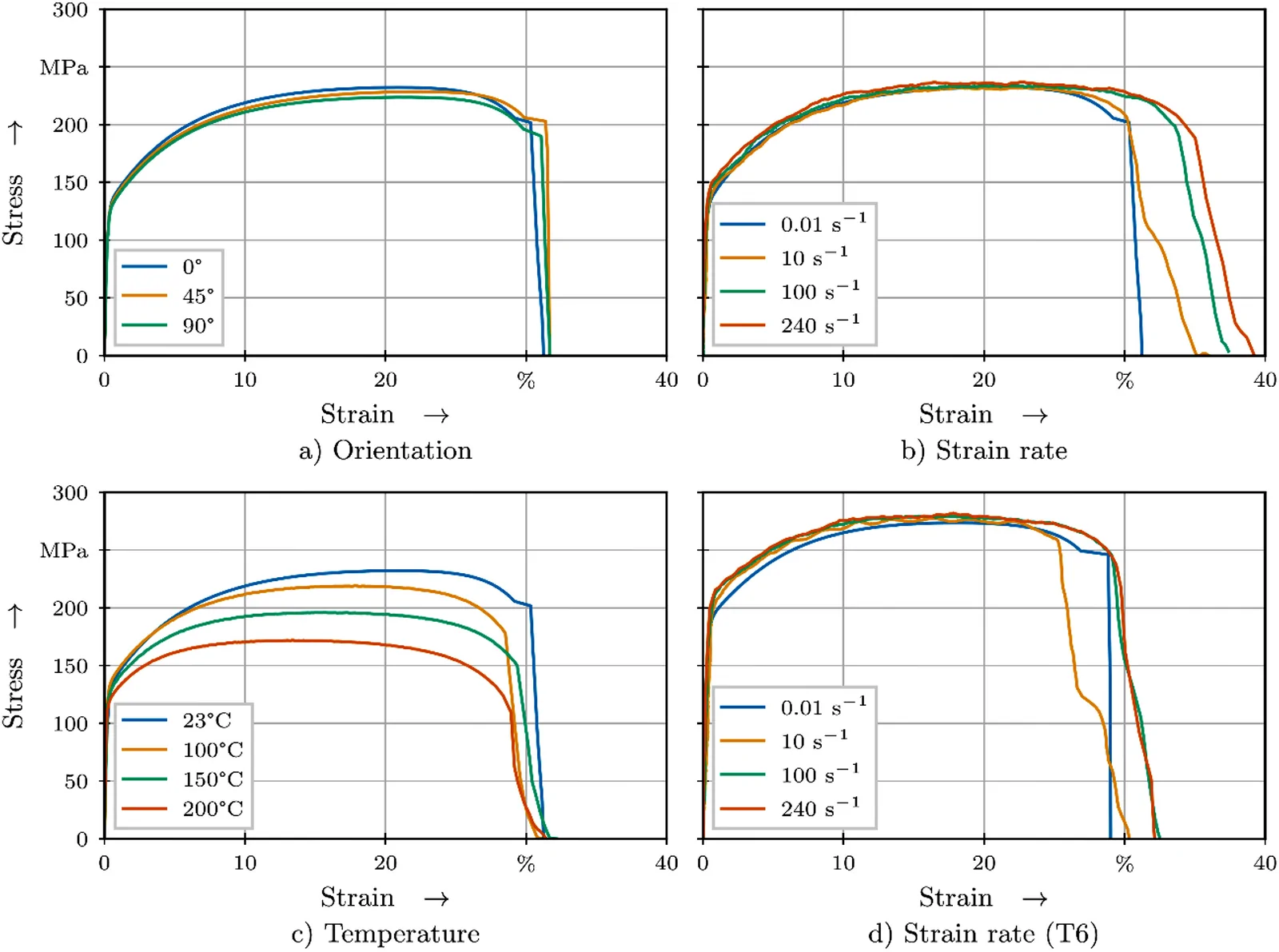
Representative stress-strain curves for different variations in testing conditions of EN-AW6014 in T4 as well as T6 condition.

For the EN-AW6014, representative stress-strain curves are shown in Fig. 3. Variations of the material in T4 state with respect to a) orientation to RD, b) strain rate and c) testing temperature as well as strain rate in T6 state are shown. At the base configuration, the aluminum initially exhibits linear behavior with a distinct transition to non-linear behavior when reaching the yield strength. From this point onward, tangent stiffness is significantly reduced. The stress gradually increases to a maximum of 230 MPa at a strain of 20.7%. From this point onward, stress slowly decreases until material failure is initiated at a strain of 30%, after which stress rapidly drops to zero. Similar to the HCT590X, no significant changes in material behavior with specimen orientation with respect to RD were observed. In general, no changes with strain rate occur, with the exception of failure progression: At quasi-static testing failure is abrupt, but becomes more and more gradual with increasing strain rate, even though the critical strain at initiation remains unchanged. On the contrary, a significant change of the non-linear stress response, ultimate stress and failure initiation with temperature is observed. Whilst linear regime and transition point remain unchanged, tangent stiffness, ultimate stress and strain at failure initiation decrease with increasing temperature. Bake hardening to achieve T6 state enlarges the linear regime. The overall non-linear behavior remains similar, with decreased tangent modulus in comparison to T4 state, leading to a reduced difference between yield and ultimate stress. Again, material behavior does not significantly change with strain rate. As opposed to the T4 state, failure becomes only slightly more progressive with increase in rate.

## 4 Experimental Design, Materials and Methods

For this investigation, tests were carried out on a dual phase steel HCT590X with a sheet thickness of 1.5 mm, with a nominal yield strength of 330 MPa and a minimum elongation of A_80_ = 20% [2]. The aluminum alloy investigated in this study is EN AW-6014 in temper T4, with a sheet thickness of 2.0 mm, a yield strength of 95 MPa, and a minimum elongation A_80_ = 25% [3]. Both are highly relevant in the automotive industry [4].

**Fig. 4:**
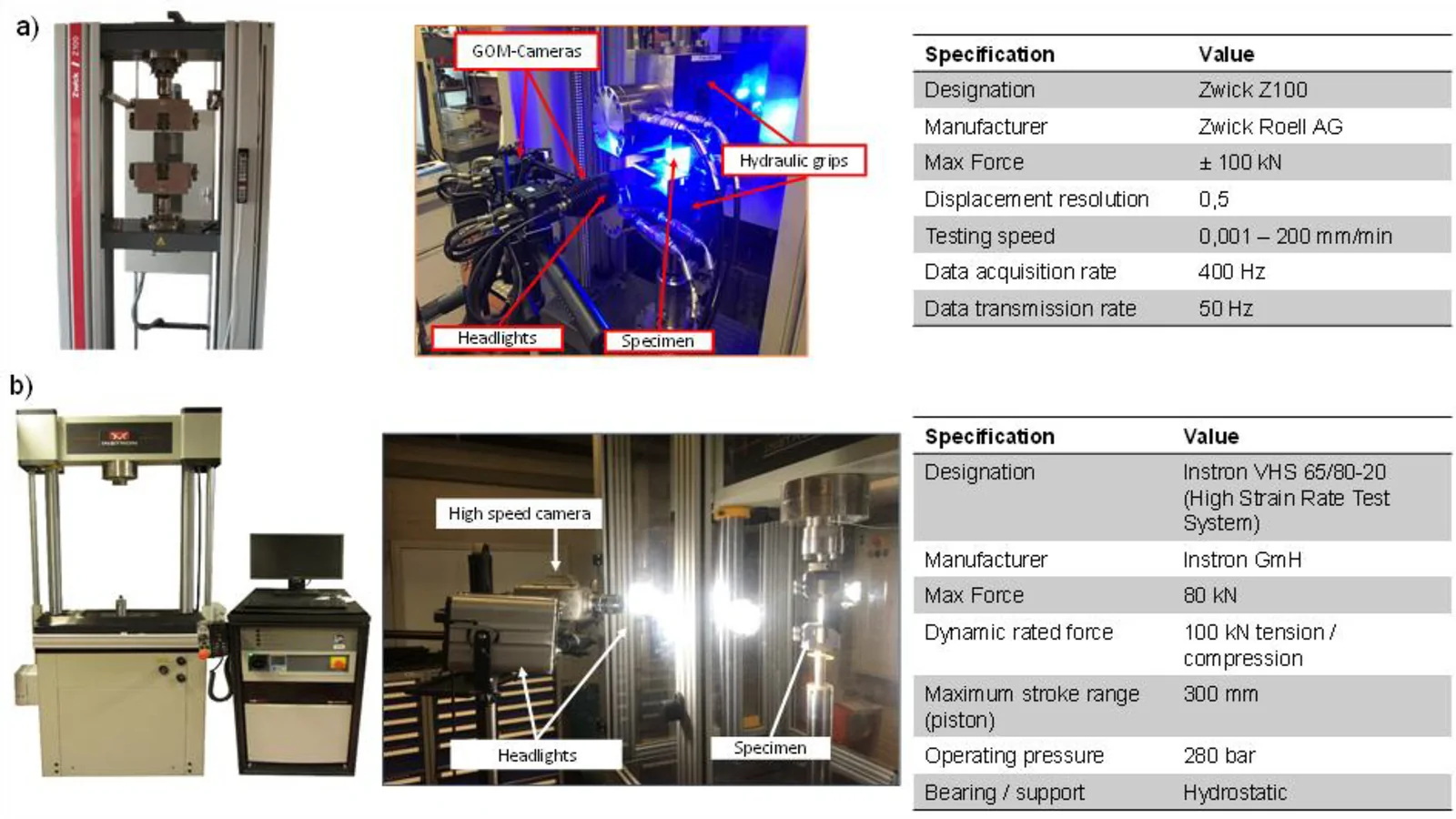
Tensile testing systems used a) Zwick Z100 and b) Instron VHS 65/80–20.

The sheet material properties were characterized using two testing systems. Quasi-static tests at strain rates of 0.001 and 0.01 s⁻¹ according to [11], as well as temperature-dependent tests following [12], were conducted on a Zwick Z100 tensile-compression testing machine. For tests at elevated temperatures, a climate chamber was integrated into the testing machine. Higher-strain-rate tests at 10 and 100 s⁻¹ were performed on an Instron VHS 65/80–20 high-speed tensile testing system. Local specimen deformation was measured optically using GOM ARAMIS in all experiments. Therefore, the reported strain was independent of crosshead displacement and did not include the compliance of the testing machine. As the achievable velocity ranges of the two testing systems did not overlap, direct cross-validation at an identical test velocity could not be performed. Fig. 4 shows the testing machines and the DIC system, together with the corresponding specifications of the Zwick Z100 and Instron VHS 65/80–20.

**Fig. 5:**
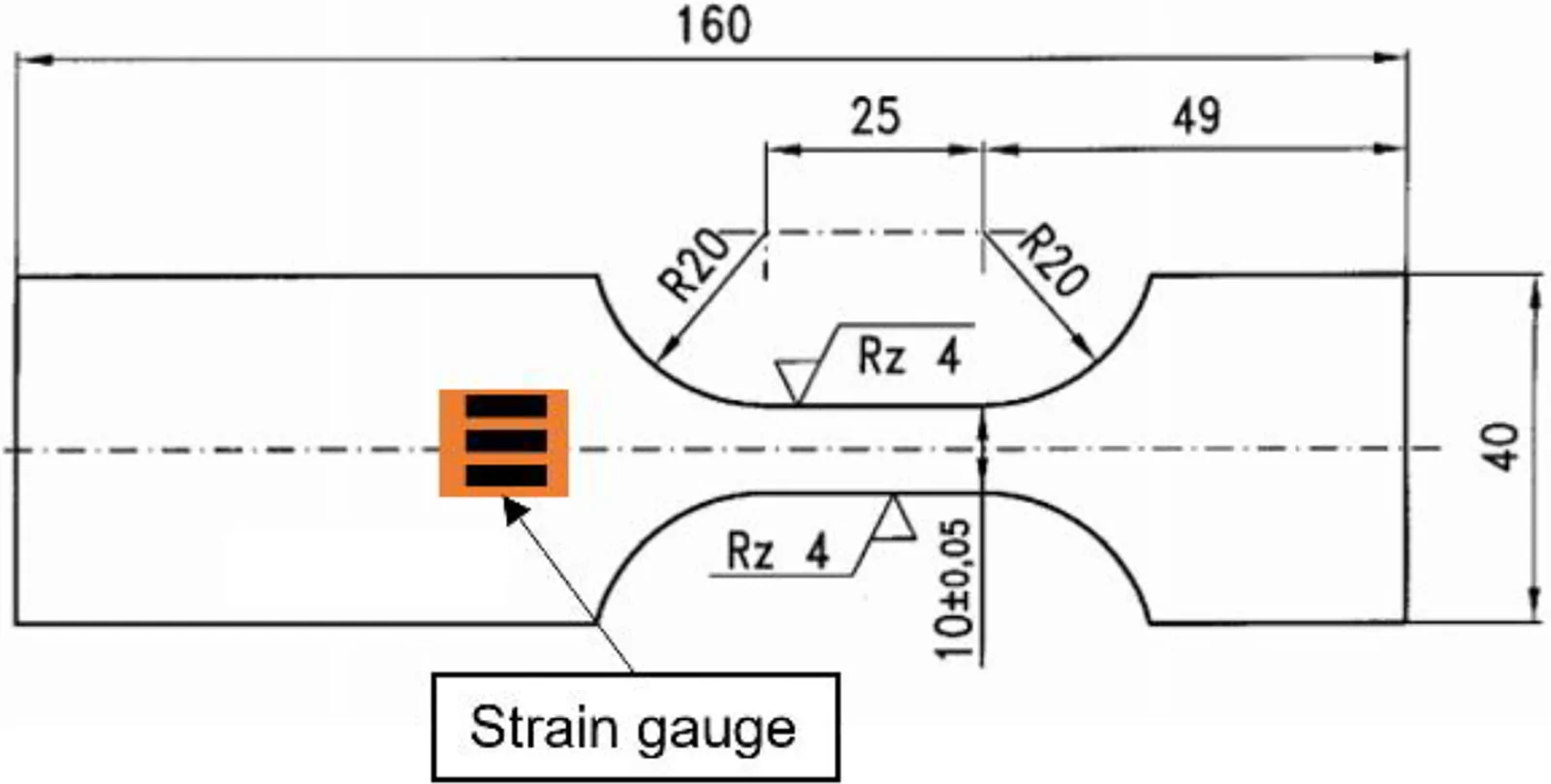
Used tensile test specimen geometry according to SEP 1230, adapted from [13].

The specimen geometry was chosen according to SEP 1230 [13], with an initial gauge length $l_{p}$ of 25 mm, to ensure comparability across all tests, as recommended in [10], and commonly applied [[14], [15], [16]]. Due to the high testing speeds, the specimens were equipped with SGD-3/120-LY41 strain gauges on the dynamometric section for dynamic force determination according to SEP 1230 [13] which is described further below. Fig. 5 shows the specimen geometry used for high-strain-rate testing together with the positions of the strain gauges. For HCT590X, additional specimens according to DIN 50,125 [17] were tested.

At high testing speeds, optical deformation measurements were performed using a GOM ARAMIS high-speed system. The system consisted of two Photron FASTCAM Nova S12 cameras with a maximum resolution of 1024 × 1024 pixels at 12,800 fps and a maximum frame rate of 1100,000 fps at reduced resolution. The images used for the local high-speed DIC evaluation were recorded at 40,000 fps with a resolution of 120 × 100 pixels, while the quasi-static tests were recorded at 15 fps. The evaluation was performed in GOM ARAMIS using a facet size of 17 pixels. A consistent evaluation template was used to determine the change in distance between two defined facet points as a virtual gauge length.

For the high-strain-rate experiments, the specimen surfaces were cleaned before the strain gauges were bonded using a liquid adhesive. The gauges were attached to both sides of the dynamometric section and connected in a bridge configuration to minimize the influence of bending moments. After mounting and preloading the specimen, the strain gauge measurement chain was connected to a signal amplifier and calibrated individually within approximately two-thirds of the elastic calibration range according to SEP 1230 [13]. Before testing, a stochastic speckle pattern was applied to the specimen surface, and the GOM ARAMIS system was calibrated for a measuring volume of 30 × 25 × 15 mm³. The force measurement and GOM ARAMIS systems were initiated using a common external trigger, providing a shared time reference for the simultaneously recorded force and deformation data. The stress-strain curves were obtained by combining the recorded force with the corresponding local DIC deformation. Engineering stress was calculated using the initial specimen cross-sectional area, while local strain was determined from the relative change in the virtual gauge length. No numerical filtering, smoothing, modulus-based correction, or manual removal of individual data points was applied. Quality control was performed at the test level: clearly unsuccessful or non-evaluable tensile tests, including tests with unusable force or DIC data, were excluded, whereas the signal histories of all accepted tests were retained without further modification.

## Limitations

A fixed set of factors were varied, almost exclusively, one at a time. This does not allow for an analysis of factor interactions.

## Ethics Statement

We confirm that the authors have read and followed the ethical requirements for publication in Data in Brief and confirm that the current work does not involve human subjects, animal experiments, or any data collected from social media platforms.

## CRediT Author Statement

**Johannes Gerritzen** Conceptualization, Writing - Original Draft, Visualization, Funding acquisition, Investigation; **Malte Schlichter** Methodology, Investigation, Data Curation, Writing - Original Draft, Investigation; **Stefan Goetz** Data Curation, Writing – Original Draft, Funding acquisition; **Robert Kupfer** Validation, Writing - Review & Editing, Funding acquisition, Visualization, Supervision, Investigation

## Data Availability

ZenodoMechanical Response Data of HCT590X and EN AW-6014 across Temperatures and Strain Rates (Original data).
